# Sex and age differences in brain metabolism and cognition in adolescents

**DOI:** 10.1162/IMAG.a.1159

**Published:** 2026-03-09

**Authors:** Anjali Balaganesh, Taylor M. Zuleger, Zexuan Liu, Jed A. Diekfuss, Jonathan A. Dudley, Weihong Yuan, Kim D. Barber Foss, Kim M. Cecil, Scott Bonnette, Gregory D. Myer, Candace C. Fleischer

**Affiliations:** Department of Biomedical Engineering, Georgia Institute of Technology and Emory University, Atlanta, GA, United States; Emory Sports Performance and Research Center (SPARC), Flowery Branch, GA, United States; Emory Sports Medicine Center, Atlanta, GA, United States; Department of Orthopaedics, Emory University School of Medicine, Atlanta, GA, United States; Department of Veterans Affairs, Atlanta VA Medical Center, Decatur, GA, United States; Department of Radiology, Cincinnati Children’s Hospital Medical Center, Cincinnati, OH, United States; Department of Radiology, University of Cincinnati College of Medicine, Cincinnati, OH, United States; Division of Sports Medicine, Cincinnati Children’s Hospital Medical Center, Cincinnati, OH, United States; Department of Radiology and Imaging Sciences, Emory University School of Medicine, Atlanta, GA, United States

**Keywords:** magnetic resonance spectroscopy, adolescence, brain development, brain metabolites, cognition

## Abstract

Adolescence is a period of neural development, marked by maturation of brain structure and function. While sex- and age-related markers of structural brain development are documented, neurochemical and cognitive changes are less understood. Our goal was to evaluate neurochemistry and cognition in adolescents as a function of sex and age. Magnetic resonance spectroscopy quantified brain metabolites, and attention networking, digital trail making, and cued task switching tests measured cognition in 354 healthy adolescents. Groupwise comparisons and linear regressions evaluated sex- and age-related effects, respectively. Males were differentiated from females in cognitive performance and brain metabolite concentrations, including myo-inositol, glutamate + glutamine (Glx), *N-*acetylaspartate, and creatine. Males performed tasks with faster speed while females demonstrated better accuracy. Decreases in Glx concentration and faster reaction times were associated with increasing age, indicative of maturing brain function during adolescence. These findings highlight adolescence as a period of active brain development.

## 1 Introduction

Adolescence is a critical period of brain development, marked by substantial biological, behavioral, and cognitive changes that shape long-term cognitive function and health (Arain et al., 2013; Dumontheil, 2016; Goddings et al., 2019). During adolescence, maturation of the brain is evidenced by increased myelination and further development of white matter tracts, increased neural connectivity, and synaptic pruning for refinement of neural networks (Corrigan et al., 2021; Giorgio et al., 2010; Lebel & Deoni, 2018; Vanes et al., 2020; Vijayakumar et al., 2016). Structural changes during adolescence often coincide with improvements in cognitive abilities, including enhanced executive function, working memory, information processing, and decision making (Grydeland et al., 2013; Seghete et al., 2013). Structural neuroimaging studies using magnetic resonance imaging (MRI) delineate a developmental trajectory, with early maturation in primary sensory and motor areas followed by gradual development in association cortices, which are responsible for higher-level cognitive function (Groeschel et al., 2010; Lu et al., 2009; Schulte et al., 2020; van Duijvenvoorde et al., 2016). Sex- and age-specific differences in neurodevelopment (Dean et al., 2015; Schulte et al., 2020; Vijayakumar et al., 2016) indicate female adolescents frequently achieve earlier peaks in white matter volumes relative to males (Asato et al., 2010; Lenroot & Giedd, 2010), a finding correlated with improved cognitive function (Schulte et al., 2020). Concurrently, gray matter reductions and cortical thinning also accompany adolescent maturation, further reflecting structural brain development (Giorgio et al., 2010).

Structural MRI studies provide valuable insights into anatomical hallmarks of brain development; however, these investigations are limited as they do not capture the neurochemical changes accompanying adolescent development. Magnetic resonance spectroscopy (MRS) offers a unique opportunity to quantify brain metabolites, providing additional information about the neurochemical basis of development (Öz et al., 2021; Zhu & Barker, 2011). Though previous work has evaluated metabolite concentrations across broad age ranges (e.g., cohorts ranging from 13 to 72 years of age, or neonates through 18 years of age) (Blüml et al., 2012; Raininko & Mattsson, 2010), and in populations with neurological or psychiatric disorders (O’Neill et al., 2004; Tandon et al., 2013) or after injury (Liu et al., 2025), the neurochemical profile of healthy adolescents remains underexplored. Mixed findings further complicate the understanding of metabolic profiles during adolescent development. Prior studies have reported age-related increases in *N*-acetylaspartate (NAA) normalized to total glycerophosphocholine + phosphocholine (tCho) (Horská et al., 2002) and NAA normalized to creatine (Ghosh et al., 2017), as well as higher glutamate + glutamine (Glu + Gln, Glx) in early adolescence and late adulthood relative to middle age (Raininko & Mattsson, 2010). In the brain, NAA serves as a marker of neuronal integrity and myelin synthesis, and Glx reflects excitatory signaling (Rae, 2014; Tsai & Coyle, 1995). Total creatine + phosphocreatine (tCr) is a marker of energy metabolism, while tCho is commonly associated with membrane turnover (Rae, 2014). Other studies have reported both increasing and decreasing concentrations of NAA and myo-inositol (mIns), a glial marker commonly associated with neuroinflammation, across childhood and adolescence (Blüml et al., 2012; Perdue et al., 2023; Rae, 2014; Yang et al., 2015). Investigations into differences in brain metabolites as a function of sex have also yielded inconsistent results, with some reports indicating no sex differences (Raininko & Mattsson, 2010) and others reporting both higher and lower concentrations of tCho in males compared to females (Ito-Shimizu et al., 2012; Kadota et al., 2001; Thomson et al., 2024). Due to the inclusion of broad age ranges, combined with mixed results on sex- and age- differences in brain metabolites, more research in larger cohorts is needed to characterize metabolite distributions in healthy adolescents.

In addition to differences in neurochemistry, prior behavioral research has documented differences between sexes during adolescence across various cognitive domains. Specifically, females often outperform males on memory, visual skills, and attention tasks, while males tend to have faster impulses and overall greater inhibitory control (Bianco et al., 2020; Mittal et al., 2012). As female adolescents often reach developmental milestones earlier than males, parallel shifts in metabolites may also underlie behavioral differences within healthy populations. Moreover, cognitive functioning follows age-related trajectories, with working memory capacity, executive function, processing speed, and pattern recognition steadily improving throughout adolescence (Anokhin et al., 2022; Fandakova et al., 2014; Ferguson et al., 2021; Heiskanen et al., 2024). Despite these well-characterized cognitive changes, few studies have investigated both brain metabolite concentrations and cognitive performance outcomes to comprehensively characterize developmental trajectories in healthy adolescent populations.

The goal of this study was to evaluate sex- and age-related differences in brain metabolite concentrations and cognition in a cohort of healthy adolescents. We hypothesized both brain metabolite concentrations and cognitive performance differ between sexes, reflecting differing maturation timelines, and hypothesized both metabolite concentrations and cognitive performance metrics would be associated with age, consistent with healthy brain development.

## 2 Materials and Methods

### 2.1 Study design

This retrospective study utilized baseline MRS data and cognitive metrics (NCT #04068883) from adolescent male and female participants (ages 13 to 18) recruited from seven high schools in the greater Cincinnati area. The trial was approved by the Institutional Review Board at Cincinnati Children’s Hospital Medical Center. Written informed consent was obtained from all participants if they were 18 years or older, or from their parents or legal guardians if they were younger than 18 years old. For those younger than 18 years old, participant assent was obtained.

**Fig. 1.**
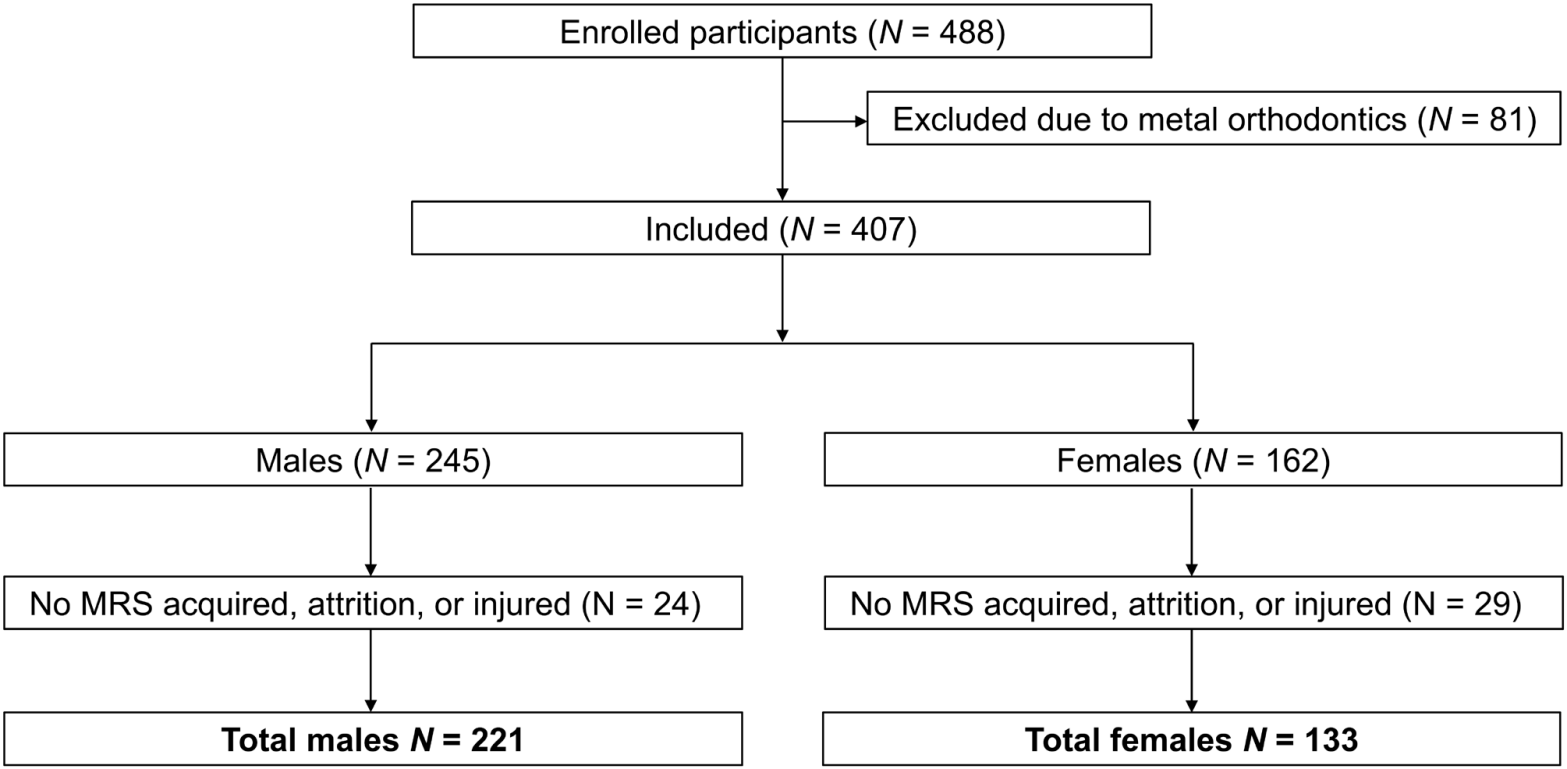
Inclusion and exclusion criteria. Following exclusion of participants due to metal contraindications to MRI, participants were included if they had MRS data acquired and were included in the prospective trial (N = 354). Of the 221 males included, 1 subject had no MRS in the left primary motor cortex (M1) and 26 subjects had no MRS in the anterior cingulate cortex (ACC). Of the 133 females, 1 subject had no MRS in the M1 region and 24 subjects had no MRS in the ACC region.

Eligibility criteria for enrollment in the study included being medically healthy and at least 13 years old. Ineligibility included a history of neurological disorders, previous cerebral infarction, severe head trauma, medical contraindications to restriction of venous outflow via jugular vein, glaucoma (normal angle or normal tension), hydrocephalus, recent penetrating brain trauma (6 months), known carotid hypersensitivity, known increased intracranial pressure, central vein thrombosis, airway obstruction, seizure disorder, prothrombotic/hyper-thrombotic condition, cerebral cavernous malformation, and/or student athletes not medically cleared to play sports. A total of 488 participants were enrolled in the study after being deemed eligible. After exclusions due to MRI contraindications, missing MRS data, attrition from the trial, or injury, 354 adolescents were included in the final analysis (Fig. 1).

### 2.2 Magnetic resonance acquisition and analysis

MR data were acquired using three 3 Tesla Phillips whole-body MRI scanners (Achieva, Ingenia, and Elition) with a 32-channel phased array head coil. Sagittal T_1_-weighted images were acquired using a 3D gradient-recalled echo sequence (repetition time [TR]/echo time [TE] = 8.1/3.7 ms, inversion time [TI] = 1070 ms, field of view [FOV] = 256 × 256 mm^2^, number of slices = 180, voxel size = 1 mm isotropic) to guide MRS voxel placement.

Single-voxel MRS data were collected in the left primary motor cortex (M1) and anterior cingulate cortex (ACC) using a ^1^H MRS point resolved spectroscopy (PRESS) sequence (TR/TE = 2000/30 ms, averages = 96, complex data points = 1024, spectral bandwidth = 2000 Hz, flip angle = 90°, nominal voxel size = 2 cm isotropic). The M1 and ACC regions were selected during prospective data collection based on the goals of the original trial. Water suppression was performed using a chemical shift selective sequence (Haase et al., 1985). A non-water-suppressed spectrum (averages = 16) was also acquired to determine absolute metabolite concentrations using the vendor-supplied water suppression sequence (Haase et al., 1985).

Metabolite concentrations, including total *N*-acetylaspartate + *N*-acetylaspartyl glutamate (tNAA), tCr, tCho, mIns, and Glx, were quantified using LCModel v6.3-1*R* (Provencher, 1993). Tissue segmentation was performed using Statistical Parametric Mapping (SPM) version 12 (SPM12, https://www.fil.ion.ucl.ac.uk/spm/). Each voxel in the T_1_-weighted image was segmented into gray matter, white matter, or cerebrospinal fluid (CSF) based on the tissue probability map in SPM12. Water-normalized metabolite concentrations from LCModel were then corrected for CSF volume and partial relaxation effects using the tissue-specific T_1_ and T_2_ relaxation times of both water (Wansapura et al., 1999) and metabolites (Traber et al., 2004), and the molar concentration was estimated as previously described (Near et al., 2021). Metabolite concentrations with Cramer-Rao lower bounds (CRLBs) ≤20% were included in the final analysis, and all concentrations are reported as millimolar (mM). Details of MRS acquisition and analysis are included in the MRSinMRS checklist (Lin et al., 2021) as an Appendix in the Supplementary Material.

### 2.3 Cognitive evaluation and analysis

#### 2.3.1 Digital trail making test

**Fig. 2.**
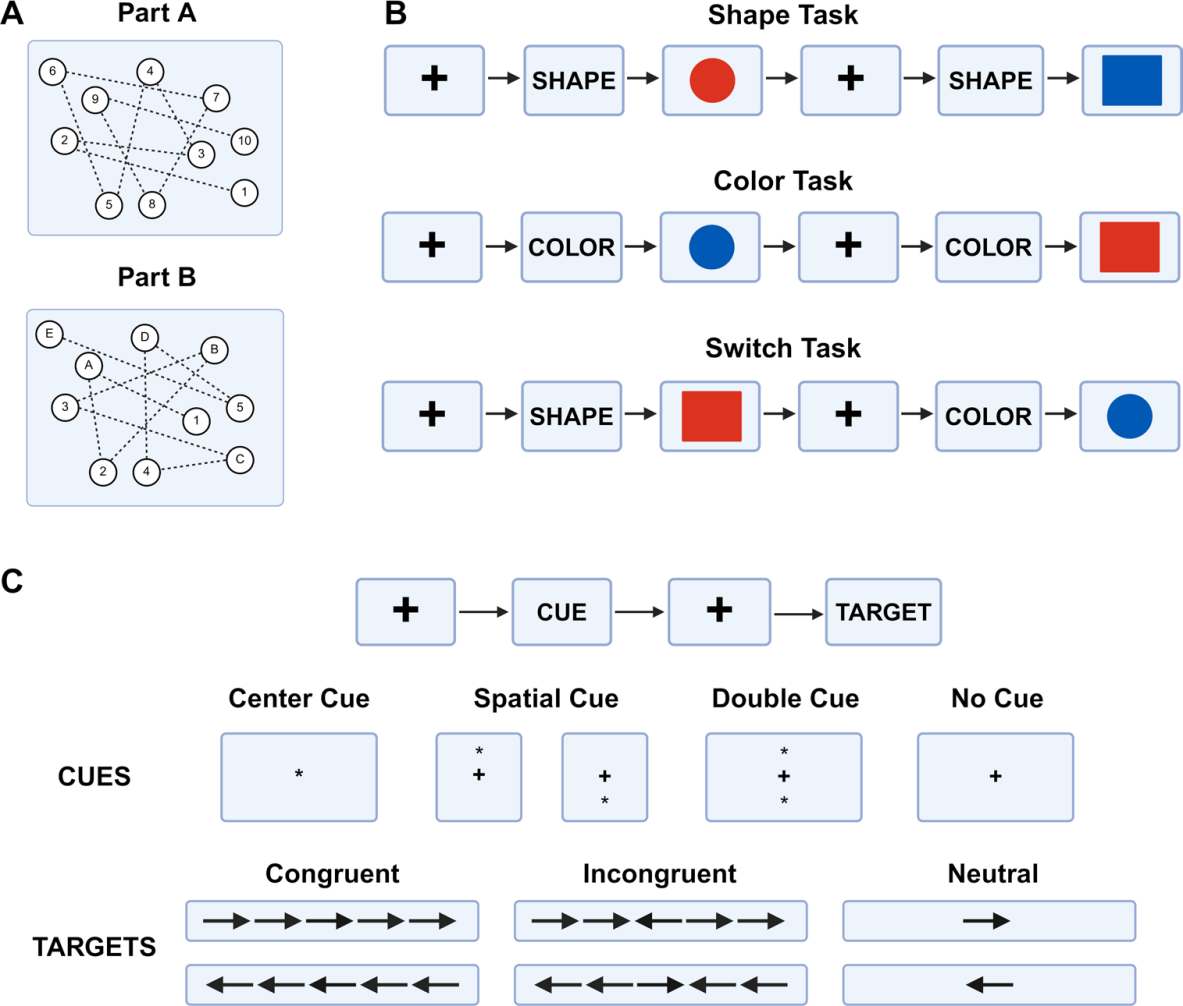
Schematic for cognitive tests*.* (A) Digital trail making test (dTMT) evaluates cognitive flexibility and executive function. Assessment is divided into two parts, A and B, and measured as the difference in reaction time (ms) of Part A subtracted from Part B. (B) Cued task switching test (cTST) measures multi-tasking and executive function, separated into three types of tasks: shape, color, and switching. Participants are assessed using multiple trials (N=12) for each task. Faster reaction time (ms) and lower error rates (fraction) indicate higher cognitive function. (C) Attention networking task (ANT) assesses selective attention. Participants are provided with a cue followed by a target. Any combination of cues and targets are possible, and the task is quantified by accuracy (on a scale of 0 to 1) and mean reaction time (ms). Higher accuracy scores and faster mean reaction times correspond with greater cognitive function (Created in BioRender. Zuleger, T. (2026) https://BioRender.com/i05o592).

The digital trail making test (dTMT) evaluated executive function and cognitive flexibility via timed connect-the-dot tasks (Fellows et al., 2017). The dTMT was administered with the Unity software (Unity Technologies) using a custom application and graphical user interface on a handheld tablet (Samsung Galaxy, S10.5) which recorded participant responses and completion times. Part A required participants to connect circles in numerical order (1-2-3-4…), while Part B required alternating numeric and alphabetic sequencing (1-A-2-B-3-C…). The difference in trial time to complete Part A relative to Part B was calculated as the primary outcome for the dTMT, with faster times indicating higher cognitive function (Fig. 2A).

#### 2.3.2 Cued task switching test

The cued task switching test (cTST) assessed multitasking and executive function by asking participants to respond to shape and color cues (Rogers & Monsell, 1995). The cTST was programmed and administered on a laptop using a custom script in E-Prime software (Psychology Software Tools). Participants pressed designated keys corresponding to either a given shape or specific color. For example, participants were asked to press the letter “n” if the shape was a circle and “k” if the shape was a square. For colors, the participants were asked to press the letter “n” if the color was red or “k” if the color was blue.

The cTST consists of three task conditions: shape-only, color-only, and switch (alternating between shape and color within a trial). If the participants were performing the shape task, they focused on determining the shape and pressing the corresponding letter on the keyboard. Similarly, if participants were performing the color task, they would only focus on determining the color and pressing the corresponding letter on the keyboard. If participants were performing the switch task, they were asked to respond to both shape and color cues within a single task. Each participant completed two repeat blocks for shape tasks, two repeat blocks for color tasks, and two switch blocks where they alternated between the shape and color tasks. Each participant completed a total of 12 trials within each block.

Switching cost and mixing cost reaction time and error rates were calculated. The switching cost reaction time (ms) was measured as the difference in mean reaction time between the correct repeat trials and switch trials within the switch blocks. Trials with reaction times less than 100 ms were excluded as they are considered anticipatory responses leading to false positives. Repeat trial error rates were calculated as the number of errors from repeat trials within switch blocks. Switch trial error rates (fraction) were calculated as the number of errors from switch trials within switch blocks. A positive switching cost reaction time indicates faster reaction time during switch trials, while a negative switching cost reaction time reflects a pattern of slower responses during switch trials. Switching cost error rate (fraction) was measured as the difference in percent error rate between repeat trials and switch trials within switch blocks. A positive switching cost error rate reflects higher error rate during switch trials, while a negative switching cost error rate fewer errors during switch trials. Mixing cost reaction time (ms) and error rates (total errors/total responses) were calculated in a similar manner, except using all trials rather than just the trials within switch blocks as used for switching cost reaction time and error rates. Faster reaction time and lower error rates for both metrics indicated higher cognitive function (Fig. 2B).

#### 2.3.3 Attention networking task

The attention networking task (ANT) assessed selective attention and inhibitory control through measures of alertness, orientation, and conflict resolution (Fan et al., 2002). The ANT was also performed using E-Prime software. Participants responded to a central flanker arrow, following one of four cue conditions (center, spatial, double, or no cue) and one of three target conditions (congruent, incongruent, and neutral). Participants pressed the left or right arrow key corresponding to the central flanker direction. Any combination of cues and targets were possible. This assessment measured executive control via average reaction time (ms) and accuracy of motor skills (scale between 0 and 1). Participants completed one experimental block of 96 trials. Average reaction time was calculated as the mean reaction time across all trials, excluding any reaction time less than 100 ms as they are considered anticipatory responses leading to false positives. Accuracy of motor skills was calculated as the fraction of correct responses across all trials. Higher accuracy scores and faster (lower) average reaction times were indicative of higher cognitive function (Fig. 2C).

### 2.4 Statistical analyses

All statistical analyses were conducted on MATLAB version R2022b (Mathworks). Age was calculated to the day using the participants’ date of birth and the date of the study visit. MR data acquisition and cognitive assessments occurred on the same day. Distributions of age, metabolite concentrations, and cognitive metrics were visually assessed. As most variables exhibited non-normal or highly skewed distributions, non-parametric tests were used for all groupwise comparisons. Outliers in metabolite concentrations and cognitive metrics were defined as values exceeding three absolute deviations from the median and were removed prior to statistical analyses. The number of subjects included for each analysis is reported within the text.

Group differences in signal-to-noise ratio (SNR) and full width at half maximum (FWHM) from LCModel, metabolite concentrations, and cognitive metrics between males and females were evaluated using Mann-Whitney U tests. False discovery rate (FDR) correction was applied to p-values for metabolite concentrations within M1 and ACC and for all cognitive metrics. FDR corrections within each voxel, but not across voxels, were performed as evaluation of metabolites within a brain region was considered most important in the simultaneous hypothesis testing approach. More subjects had MRS data in M1 compared to ACC, further motivating the less conservative approach taken for hypothesis testing in this observational study. Univariate linear regressions were performed for each metabolite concentration in each voxel (response) as a function of age (predictor), sex (covariate), scanner (covariate), and the interaction effect between age × sex. Regressions using metabolite concentrations were also performed without scanner as a covariate to evaluate if any of the effects are driven by MR scanner. Similarly, separate univariate linear regressions were conducted for each cognitive metric (response) as a function of age (predictor), sex (covariate), and interaction effect between age × sex. For significant age × sex interactions, post-hoc regressions between either cognitive metric or metabolite concentration (response) and age for each group (males or females) were conducted. Exploratory regressions were also conducted to evaluate relationships between metabolite concentrations and cognitive metrics. Specifically, cognitive metrics (response) and metabolite concentrations (predictor) significantly associated with age in the primary analysis were evaluated using separate linear regression models, with age included as a covariate. Statistical significance was determined by p ≤ 0.05 for all analyses. Effect sizes were reported for significant p-values using Cliff’s delta (δ) (Kane & Esther, 2024) for groupwise comparisons and *r* for correlations. Absolute δ values are reported, and direction is indicated in the text. Effect sizes (δ or *r*) were classified as small, medium, and large as previously reported for δ (Kane & Esther, 2024) and *r* (Brydges, 2019).

## 3 Results

**Fig. 3.**
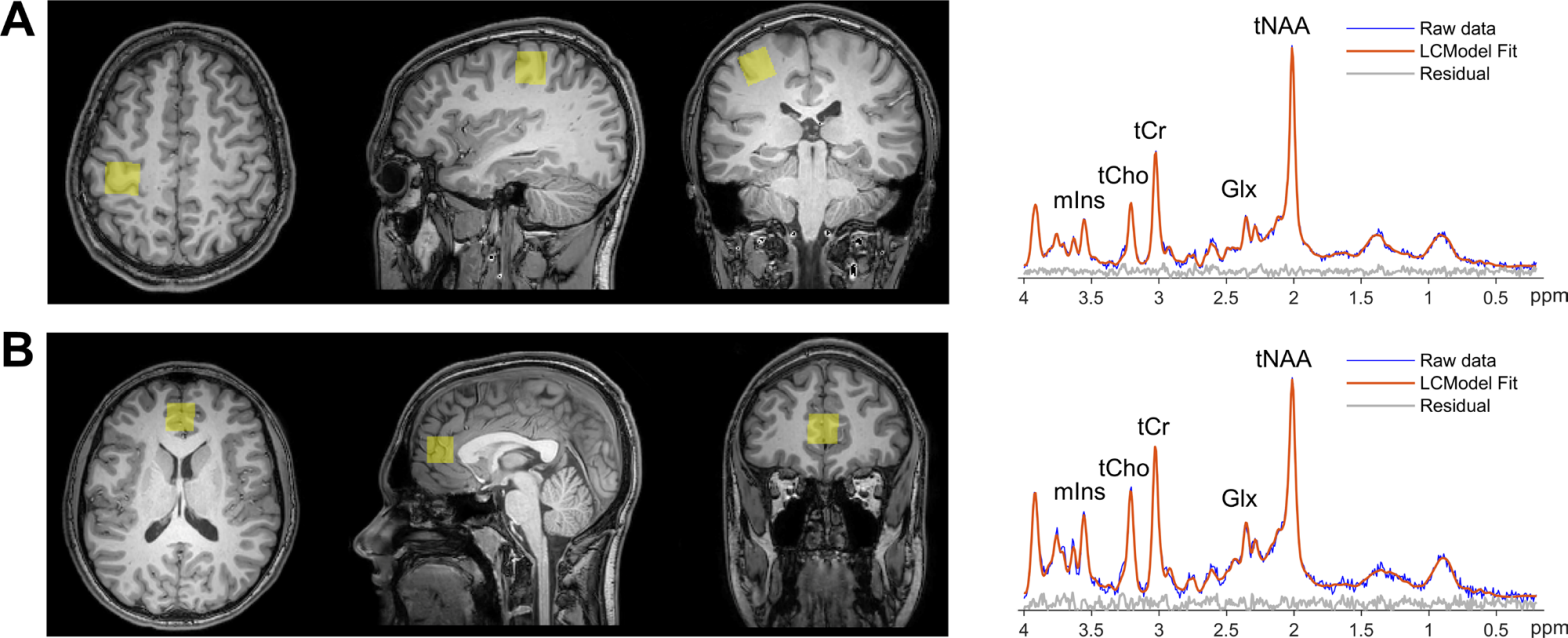
In vivo magnetic resonance spectroscopy (MRS) voxel positioning and corresponding spectra. Representative MRS voxel position (yellow square) and the corresponding raw and fitted spectra acquired in (A) left primary motor cortex and (B) anterior cingulate cortex. Voxel positions are overlaid on T_1_-weighted images and displayed in neurological orientation for a 17-year-old male participant. (tNAA = total *N*-acetylaspartate + *N*-acetylaspartyl glutamate, tCr = total creatine + phosphocreatine, tCho = total glycerophosphocholine + phosphocholine, mIns = myo-inositol, Glx = glutamine + glutamate).

A total of 354 adolescents were included in the final cohort, 221 males and 133 females (Table 1). Representative voxel positioning and in vivo MR spectra acquired from the M1 and ACC regions are shown in Figure 3. No metabolites for any voxel or subject were excluded due to CRLBs. Median SNR for spectra acquired in M1 was 34 [33–37] in males and 35 [33–37] in females. The median FWHM in M1 was 0.04 [0.03–0.04] ppm in males and 0.04 [0.03–0.04] ppm in females. Neither SNR (Z = -1.61, p = 0.21) nor FWHM (Z = 1.0, p = 0.32) for spectra acquired in M1 differed significantly as a function of sex. For the ACC, median SNR in ACC was 23 [19–26] in males and 26 [21–28] in females. The median FWHM in ACC was 0.05 [0.04–0.05] ppm in males and 0.04 [0.03–0.05] ppm in females. In females, SNR was significantly higher in spectra acquired in the ACC (Z = -3.47, p = 0.001) while FWHM was significantly lower compared to males (Z = 2.67, p = 0.008). Males were significantly older than females (Z = 4.38, p < 0.0001), with a median difference of 0.7 years. Histograms of participant ages are presented in Supplementary Figure S1.

**Table 1.** Demographic table for final cohort.

	Sample size (N,%)	Age (median [IQR] years)
All participants	354	16.2 [15.5–17.2]
Males	221 (62%)	16.5 [15.7–17.4]
Females	133 (38%)	15.8 [15.1–16.9]

IQR = interquartile range.

### 3.1 Differences in brain metabolite concentrations and cognitive performance as a function of sex

**Fig. 4.**
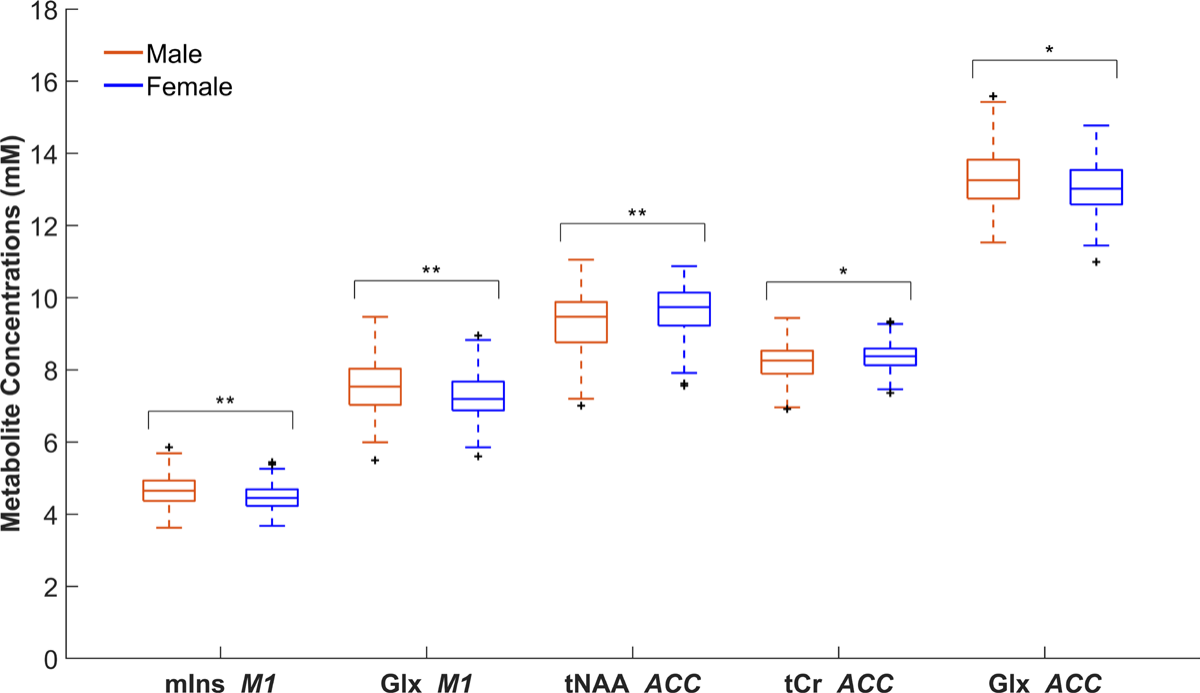
Differences in brain metabolite concentrations between male and female adolescents*.* Box plots depict sex-related differences in brain metabolite concentrations. Males had significantly higher concentrations of mIns in left primary motor cortex (M1) *(*p_FDR_ = 0.001, δ = 0.24, *N_male_* = 220, *N_female_* = 129), Glx in M1 *(*p_FDR_ = 0.003, δ = 0.21, *N_male_* = 219, *N_female_* = 128), and Glx in the anterior cingulate cortex (ACC) *(*p_FDR_ = 0.02, δ *=* 0.17, *N_male_* = 192, *N_female_* = 108). Females had significantly higher concentrations of tNAA *(*p_FDR_ = 0.003, δ = 0.24, *N_male_* = 187, *N_female_* = 105) and tCr in the ACC *(*p_FDR_ = 0.02, δ = 0.19, *N_male_* = 191, *N_female_* = 105) compared to males. Plus signs (+) indicate outliers, more than 1.5× higher or lower than the interquartile range (IQR). *p ≤ 0.05, **p ≤ 0.01. (tNAA = total *N*-acetylaspartate + *N*-acetylaspartyl glutamate, tCr = total creatine + phosphocreatine, mIns = myo-inositol, Glx = glutamine + glutamate, tCho = total glycerophosphocholine + phosphocholine).

Histograms of all brain metabolite concentrations in M1 and the ACC for both males and females are shown in Supplementary Figures S2 and S3, respectively. Groupwise comparisons of brain metabolite concentrations revealed significant differences for some metabolites between males and females in both M1 and the ACC (Fig. 4, Supplementary Table S1). In M1, males exhibited significantly higher concentrations of mIns (Z = 3.73, (FDR)-corrected p-value [p_FDR_] = 0.001, δ = 0.24) and Glx (Z = 3.23, p_FDR_ = 0.003, δ = 0.21) when compared to females. In the ACC, males had significantly higher concentrations of Glx (Z = 2.49*,* p_FDR_ = 0.02, δ = 0.17) compared to females, while females had significantly higher concentrations of tNAA (Z = -3.45, p_FDR_ = 0.003, δ = 0.24) and tCr (Z = -2.72, p_FDR_ = 0.02, δ = 0.19) compared to males. Medium effect sizes were observed for all significant groupwise comparisons of metabolite concentrations. No significant differences in tNAA, tCr, or tCho in M1 or tCho and mIns in the ACC were observed between males and females.

**Fig. 5.**
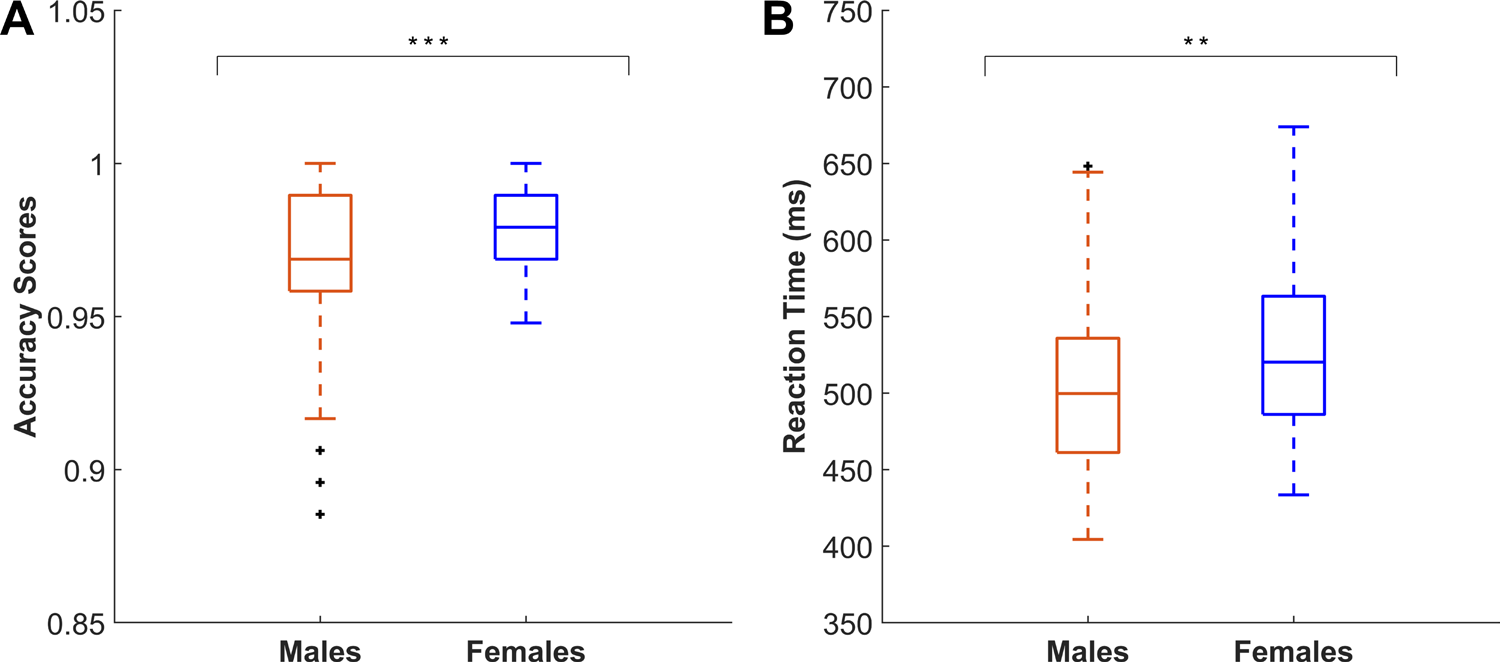
Differences in cognitive metrics between male and female adolescents. (A) Higher accuracy scores on the attention networking task (ANT) were observed for females compared to males (p_FDR_ < 0.0001, δ = 0.31, *N_male_* = 209, *N_female_* = 128). Accuracy scores range from 0 to 1, with 1 being the highest. (B) Faster reaction time measured during the ANT were observed for males compared to females (p_FDR_ = 0.0002, δ = 0.26, *N_male_* = 218, *N_female_* = 130). Plus signs (+) indicate outliers, more than 1.5× higher or lower than the interquartile range (IQR). **p ≤ 0.001, ***p ≤ 0.0001).

Cognitive assessments revealed males exhibited lower accuracy scores on the ANT (Z = -4.86*,* p_FDR_ < 0.0001, δ = 0.31) but faster average reaction time (Z = -4.00*,* p_FDR_ = 0.0002, δ = 0.26) relative to females (Fig. 5, Supplementary Table S2). Medium effect sizes were similarly observed for all significant groupwise comparisons of cognitive metrics. No significant differences between males and females were observed in performance on the dTMT or the cTST.

### 3.2 Associations of brain metabolite concentrations and cognition with age

**Fig. 6.**
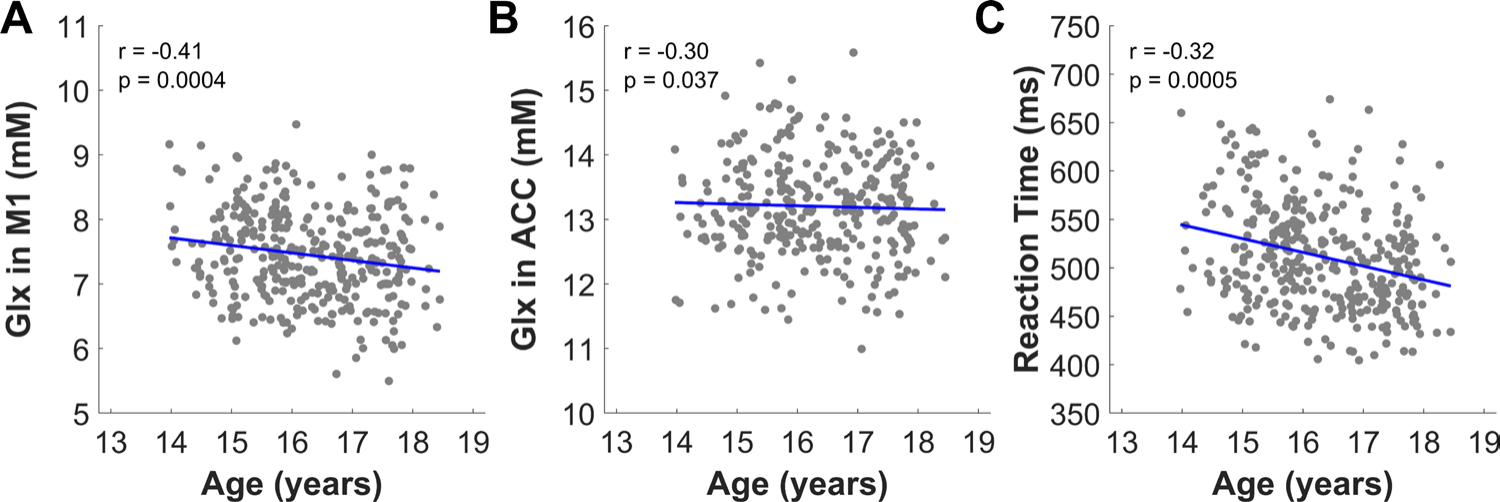
Significant associations between brain metabolite concentrations and cognitive performance with age. Brain metabolites were negatively associated with age, including (A) Glx in left primary motor cortex (M1) (p = 0.0004, *r* = -0.41, *N_male_* = 219, *N_female_* = 128) and (B) Glx in anterior cingulate cortex (ACC) (p = 0.037, *r* = -0.30, *N_male_* = 192, *N_female_* = 108). (C) Reaction times measured during the attention networking task (ANT) were negatively associated with age (p = 0.0005, *r* = -0.32, *N_male_* = 218, *N_female_* = 130). Linear correlation coefficients (*r*), which is a measure of the effect size, along with associated p-values, are reported on each plot.

Metabolite concentrations in both brain regions were significantly associated with age (Fig. 6A, B). Specifically, Glx in M1 (β = -0.15, t = -3.58, p = 0.0004, *r* = -0.41) and Glx in the ACC (β = -0.11, t = -2.09, p = 0.037, *r* = -0.30) were negatively associated with age. Regressions performed without scanner as a covariate revealed similar results, suggesting the findings are not driven by the MR scanner used to acquire MR spectra. Medium to high effect sizes were observed for associations of Glx with age. No significant associations were observed between tNAA, tCr, tCho, and mIns in M1 or ACC and age.

Cognitive assessments revealed average reaction time during the ANT was negatively associated with age (β= -12.14, t = -3.52, p = 0.0005, *r* = -0.32), indicating improved performance with age during adolescence (Fig. 6C). Significant age × sex interaction effects were observed for cTST mixing cost error rate (β = 0.02, t = 2.02, p = 0.04). Post-hoc regressions showed cTST mixing cost error significantly increased with age for females (β = 3.42, t = 2.71, p = 0.008) but not males (β = 0.22, t = 0.21, p = 0.83). The dTMT trial times and cTST reaction times were not significantly associated with age. Exploratory analyses showed no correlation between average ANT reaction time as a function of brain metabolites (Glx in M1: β = 0.82, t = 0.20, p = 0.84, Glx in ACC: β = 0.006, t = 0.0015, p = 0.99). Age remained the only significant predictor when included in the model (Glx in M1: β = -13.7, t = -4.99, p < 0.0001, Glx in ACC: β = -15.52, t = -5.33, p < 0.0001).

## 4 Discussion

In the present study, MRS was used to investigate metabolite differences in M1 and ACC in a cohort of healthy adolescents. Our findings revealed sex- and age-related differences in metabolite concentrations and cognitive performance, which may reflect distinct neurodevelopmental trajectories. Specifically, males demonstrated higher concentrations of mIns and Glx in M1, and Glx in the ACC, compared to females. Females demonstrated higher concentrations of tNAA and tCr in the ACC compared to males. Cognitively, males demonstrated faster reaction time and lower accuracy scores, while females exhibited relatively higher accuracy. Additionally, Glx concentrations in both brain regions and ANT reaction time decreased with age in both sexes.

Previous MRS findings are mixed regarding sex-related differences in metabolite concentrations. We observed higher mIns concentrations in males compared to females, consistent with prior studies of children and adolescent (6–15 years) (Cichocka et al., 2018). As mIns is considered to be a glial cell marker and osmolyte related to development, oxidative stress, and neuroinflammatory response (Haris et al., 2011), these differences likely reflect distinct developmental trajectories between males and females. While further studies are needed to understand mechanistic differences, higher mIns in males may indicate higher glial cell density. Additionally, prior reports show varied results regarding differences in Glu or Glx as a function of sex (Hädel et al., 2013; Sailasuta et al., 2008). Differences in excitatory signaling or metabolic activity may explain these collective observations, as prior studies have shown development of excitatory signaling as well as microstructural brain changes during childhood and adolescence (Norbom et al., 2021; Sorrells et al., 2019). Higher concentrations of Glx in males may reflect increased excitatory signaling compared to females (Norbom et al., 2021; Sorrells et al., 2019). The male participants in our cohort were older than the female participants and, given males typically develop later than females, some of our results may be confounded by this age difference.

Male and female adolescents differ in their trajectories of brain development, with females typically exhibiting earlier maturation of white matter pathways (Kaczkurkin et al., 2019; Lenroot & Giedd, 2010). In the present study, females demonstrated higher tNAA and tCr concentrations in the ACC relative to males, consistent with prior research reporting higher tNAA concentrations in young females relative to males in a cohort aged 6 to 15 years old (Cichocka et al., 2018). As tNAA is a marker of neuronal integrity and closely linked to white matter maturation (Horská et al., 2002), elevated concentrations of tNAA in female adolescents relative to males may indicate more advanced neuronal development (i.e., differences in myelination, neural circuits, and white matter volumes) (Lenroot & Giedd, 2010; Vijayakumar et al., 2016). Additionally, we also observed higher tCr concentration in females relative to males, consistent with a prior study reporting higher concentrations of creatine in female compared to male adults (20–59 years) (Ito-Shimizu et al., 2012). In comparison, others have reported tCr concentrations are greater in males in childhood and early adolescence compared to females (6–15 years) (Cichocka et al., 2018). Together, prior data indicate tCr varies throughout development and differ between sexes; however, many MRS studies use tCr as a reference metabolite (Wilson et al., 2019) leaving tCr relatively underexplored. Our observations of neurochemical differences between healthy male and female adolescents may be indicative of sex-specific differences in developmental timelines.

Cognitive metrics of reaction time and accuracy scores on the ANT also differed between males and females, broadly consistent with prior literature indicating males emphasize speed over accuracy (Bianco et al., 2020; Katić et al., 2012). Fitts’ Law classically described the speed-accuracy tradeoff in movement tasks that require aiming, whereby movement time increases (speed is slower) as targets increase in distance and decrease in size (more difficult to maintain accuracy). This phenomenon of increasing speed at the expense of accuracy or vice versa has since been extended to a wide range of concepts, including decision making, decision thresholds, and motor control, with numerous sex-related factors contributing to tradeoff management (Brogmus, 1991; Drugowitsch et al., 2015; Fitts, 1954; Raisbeck et al., 2020). Our findings align with sex-based studies demonstrating females tend to prioritize accuracy whereas males tend to prioritize speed (Bianco et al., 2020; Dorfberger et al., 2009; Katić et al., 2012). Though we did not directly manipulate speed-accuracy task constraints within the cognitive tests, our findings may point to sex-differences in cognitive control, with males potentially exhibiting poorer inhibitory control compared to females (Bianco et al., 2020; Schulte et al., 2020). The current findings may reflect conscious or unconscious tradeoff strategies, where males prioritize speed and females prioritize accuracy. It is also possible prior sex-specific experiences in speed-accuracy trade-off scenarios may have contributed to the perceived goal of the ANT. Additionally, underlying biological factors may have influenced the speed-accuracy trade-off observed between sexes. Females experience puberty earlier than males, one potential reason for our observation of higher accuracy rates in cognitive tasks compared to males (Schulte et al., 2020). Prior work has also suggested male adolescents are prone to more impulsivity (Laube et al., 2020), which may result in prioritization of speed during cognitive tasks over accuracy. Faster reaction times observed in males during the ANT may be explained by prior observations that adolescent males typically participate in more physical activities than females (Rosselli et al., 2020). Lastly, as development often occurs more rapidly in young females (Lenroot & Giedd, 2010), these results may reflect sex-specific differences in motor development, executive function, and inhibitory control during adolescence.

In addition to sex-related differences, we observed negative associations between Glx concentrations in M1 and ACC and age. Notably, these associations were not moderated by sex (age x sex interaction effects were not significant). Previous studies have reported a decrease in Glx beginning at late childhood through early adolescence (7–11 years) (Perdue et al., 2023) and in cohorts spanning ages 5 to 35 years (Thomson et al., 2024) and 8 to 24 years (Volk et al., 2019). Glu plays an important role in excitatory signaling (Bezalel et al., 2019; Steel et al., 2020), and the observed associations of age and Glx may reflect changes in neural circuitry and strengthening of neurotransmission (Sorrells et al., 2019). In support of these potential changes in neural transmission, our results also revealed faster reaction time with increasing age for both sexes. These results are in line with prior work showing slower reaction time during early childhood and gradual improvement during adolescence (Bucsuházy & Semela, 2017; Heiskanen et al., 2024). Additionally, our results showed the association between cTST mixing cost error rate and age differed between males and females (significant age x sex interaction effect). As age increased, lower mixing cost error rates between tasks were observed in females but not males. These results may reflect females maturing faster than males due to both structural and functional differences. Our exploratory analyses revealed changes in cognitive function and metabolite concentrations are primarily driven by age rather than directly affecting each other. Cumulatively, the data indicate adolescence is an active period of brain development marked by both neurochemical and functional maturation, whereby improvements in neural transmission may reflect more efficient processing, which, in turn, supports improvements in cognitive performance.

While this study represents one of the largest homogenous populations of healthy adolescents, particularly among studies with brain MRS, we acknowledge the following limitations in interpretation of the results. First and importantly, we acknowledge the relatively small demographic and geographic region from where all participants were recruited (greater Cincinnati area) limits generalizability to global adolescent populations. T_1_ and T_2_ times for adult populations were used to correct for partial relaxation effects on metabolite quantification. Prior studies have shown differences in relaxation times between adolescents and adults, reflecting longer T_2_ times in adult populations (40 ± 18 years) (Traber et al., 2004) compared to pediatric populations (0–4 years) (Leppert et al., 2009). As adolescent T_1_ and T_2_ times are currently unreported, this is a future area of investigation that should be considered. Additionally, previous studies were in mixed agreement compared to the results in the current manuscript. As many prior reports did not correct for T_1_ and T_2_ relaxation times, this may be one explanation for the differences. Two brain regions were used for evaluation as they are related to motor control and cognition (M1 and ACC, respectively), and characterization of brain metabolites in additional brain regions may be warranted. Our sample included a larger number of male participants relative to females, and the older mean age of the male cohort may have influenced groupwise comparisons. While differences in cognitive metrics were consistent with prior studies reporting differences in development between males and females, it is possible the observed differences in metabolite concentrations between sexes are simply due to an older male cohort (median difference of 0.7 years older than females). While small variations between sexes were observed in spectral metrics acquired in the ACC, these are unlikely the reason for the observed differences in metabolite concentrations. We acknowledge the cognitive assessments were limited to a subset of neuropsychological parameters and largely focused on cognitive-motor performance. Gross measures of motor control (e.g., balance, gait) may also be warranted in future studies to elucidate the relationships between these measures and metabolic concentrations in M1. Finally, we recognize the limitations of cross-sectional investigations, specifically the difficulty to interpret trends across age and biological sex from data acquired at a singular time point. Future longitudinal studies are necessary to fully characterize the trajectory of neurochemistry and cognitive abilities throughout adolescence.

In conclusion, we identified significant sex- and age-related differences in brain metabolite concentrations and cognition among healthy adolescents. Males and females exhibited differential brain metabolite concentrations in M1 and ACC, which may represent broader sex-differences in neurological function. Males also demonstrated faster reaction times; however, females exhibited greater accuracy on cognitive tasks which indicate a speed-accuracy tradeoff between sexes. Cognitive results indicate sex differences in cognitive control or reflect the adoption of sex-specific decision making strategies to accomplish the tasks. The observed relationships between Glx and improved reaction time with age reflect adolescence as an active period of healthy brain development. Our results highlight the importance of examining both age and sex as covariates in future brain MRS and MRI studies, particularly in adolescent populations, to better elucidate healthy brain development.

## Supplementary Material

Supplementary Material

## Data Availability

Compiled data used to perform the analyses will be made available upon reasonable request to the corresponding author and after a data-sharing agreement is executed. No original code was developed to conduct this study other than to perform standard statistical analysis.
