# Supplementary material for: Sex and age differences in brain metabolism and cognition in adolescents
Source: Imaging Neurosci (Camb). 2026 Mar 9;4:IMAG.a.1159. doi: 10.1162/IMAG.a.1159 (PMC12973075; doi:10.1162/IMAG.a.1159)
Supplement: Supplementary Material [file IMAG.a.1159_supp.pdf]

Sex and age differences in brain metabolism and cognition in adolescents

Supplementary Information

Anjali Balaganesh[1], Taylor M. Zuleger[2,3,4,5], Zexuan Liu[1], Jed A. Diekfuss[2,3,4,5], Jonathan A. Dudley[6], Weihong Yuan[6,7], Kim D. Barber Foss[2,3,4], Kim M. Cecil[6,7], Scott Bonnette[8], Gregory D. Myer[2,3,4,5], Candace C. Fleischer[1,9,*]

[1]Department of Biomedical Engineering, Georgia Institute of Technology and Emory University, Atlanta, GA, USA
[2]Emory Sports Performance and Research Center (SPARC), Flowery Branch, GA, USA
[3]Emory Sports Medicine Center, Atlanta, GA, USA
[4]Department of Orthopaedics, Emory University School of Medicine, Atlanta, GA, USA
[5]Department of Veterans Affairs, Atlanta VA Medical Center, Decatur, GA, USA
[6]Department of Radiology, Cincinnati Children's Hospital Medical Center, Cincinnati, OH, USA
[7]Department of Radiology, University of Cincinnati College of Medicine, Cincinnati, OH, USA
[8]Division of Sports Medicine, Cincinnati Children's Hospital Medical Center, Cincinnati, OH, USA
[9]Department of Radiology and Imaging Sciences, Emory University School of Medicine, Atlanta, GA, USA

*To whom correspondence should be addressed:
Candace Fleischer
1750 Haygood Drive
Atlanta, GA 30322
candace.fleischer@emory.edu

File includes:

Supplementary Figure 1

Supplementary Figure 2

Supplementary Figure 3

Supplementary Table 1

Supplementary Table 2

Appendix

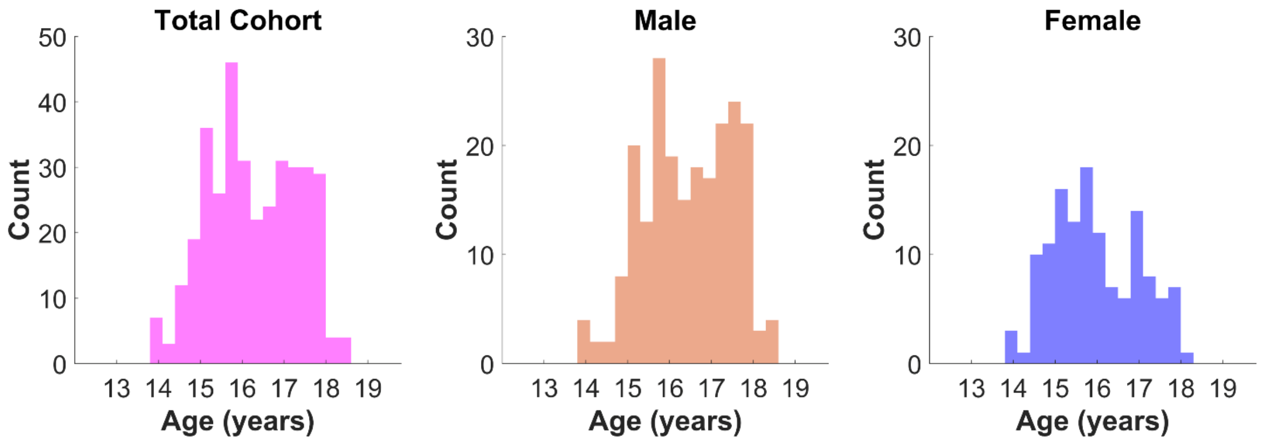

Supplementary Figure 1. Age distributions for all participants (*N* = 354) and separately for males (*N* = 221) and females (*N* = 133).

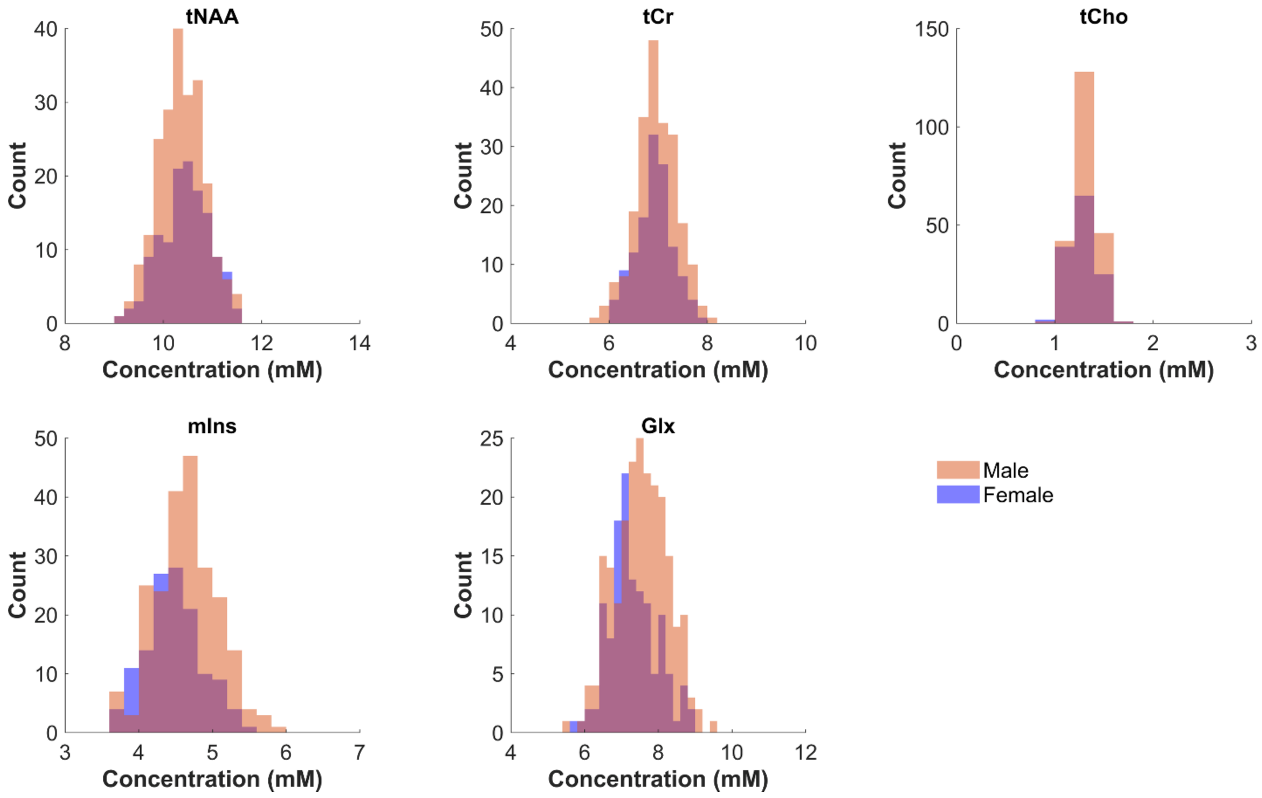

Supplementary Figure 2. Distributions of brain metabolites measured in the left primary motor cortex (M1) across all participants and denoted by sex. (tNAA = total *N*-acetylaspartate + *N*-acetylaspartyl glutamate, tCr = total creatine + phosphocreatine, tCho = total glycerophosphocholine + phosphocholine, mIns = myo-inositol, Glx = glutamine + glutamate)

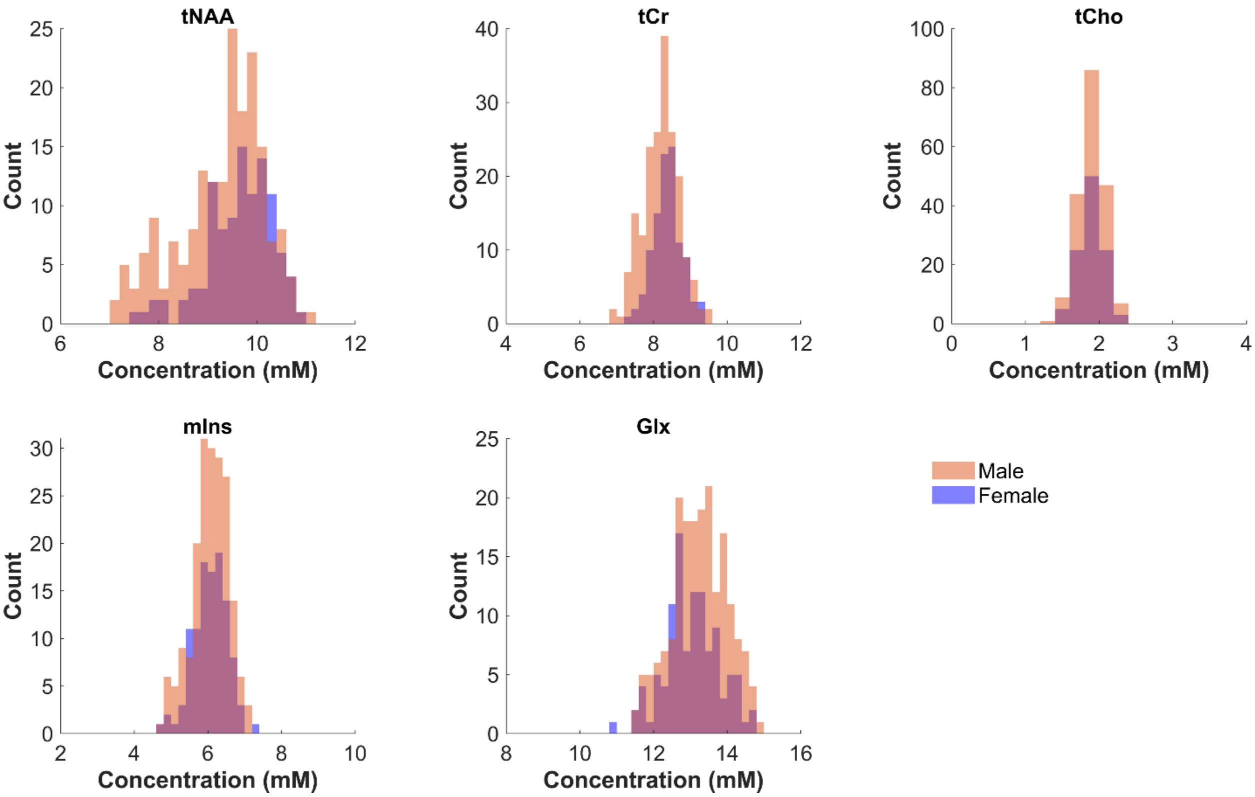

Supplementary Figure 3. Distributions of brain metabolites measured in the anterior cingulate cortex (ACC) across all participants and denoted by sex (tNAA = total *N*-acetylaspartate + *N*-acetylaspartyl glutamate, tCr = total creatine + phosphocreatine, tCho = total glycerophosphocholine + phosphocholine, mIns = myo-inositol, Glx = glutamine + glutamate)

Supplementary Table 1. Differences in brain metabolite concentrations between males and females.

**Region**	**Metabolite**	**Males (mM)**	**Females (mM)**	***Z***	**p_{FDR}**
**M1**	tNAA	10.4 [10.1 - 10.7]	10.5 [10.1 - 10.8]	-1.7	0.1
	tCr	7.0 [6.7 - 7.2]	6.9 [6.7 - 7.1]	0.7	0.5
	tCho	1.3 [1.2 - 1.4]	1.3 [1.2 - 1.4]	2.1	0.06
	mIns	4.7 [4.4 - 4.9]	4.5 [4.2 - 4.7]	3.7	**0.001**
	Glx	7.5 [7.0 - 8.0]	7.2 [6.9 - 7.7]	3.2	**0.003**
**ACC**	tNAA	9.5 [8.8 - 9.9]	9.7 [9.2 - 10.1]	-3.4	**0.003**
	tCr	8.3 [7.9 - 8.5]	8.4 [8.1 - 8.6]	-2.7	**0.02**
	tCho	1.9 [1.8 - 2.0]	1.9 [1.8 - 2.0]	1.0	0.4
	mIns	6.1 [5.8 - 6.4]	6.1 [5.8 - 6.4]	0.3	0.8
	Glx	13.3 [12.7 - 13.8]	13.0 [12.6 - 13.5]	2.5	**0.02**

Metabolite are reported as median [interquartile range]. Mann-Whitney U tests were used to compare concentrations between males and females, and Z-statistics are reported. Significant FDR-corrected p-values are bolded. FDR = false discovery rate, M1 = left primary motor cortex, ACC = anterior cingulate cortex, tNAA = total *N*-acetylaspartate + *N*-acetylaspartyl glutamate, tCr = total creatine + phosphocreatine, tCho = total glycerophosphocholine + phosphocholine, mIns = myo-inositol, Glx = glutamine + glutamate.

Supplementary Table 2. Differences in cognitive performance between males and females.

Cognitive test	Metric	Male	Female	*Z*	p_{FDR}
**dTMT**	Reaction time (ms)	18.2 [14.4 - 24.9]	18.3 [13.8 - 24.2]	0.44	0.83
**cTST**	Switching cost reaction time (ms)	-2.16 [-57.0 - 38.5]	-2.61 [-41.2 – 37.2]	-0.37	0.83
	Mixing cost reaction time (ms)	116.7 [71.5 – 162.9]	116.7 [66.4 – 176.3]	-0.13	0.89
	Switching Cost error rate	-0.05 [-0.09 - 0]	-0.05 [-0.09 - 0]	0.40	0.83
	Mixing Cost error rate	-0.06 [-0.10 - 0.02]	-0.06 [-0.08 - 0.02]	0.44	0.83
**ANT**	Average RT (ms)	499.8 [461.2 – 535.8]	520.2 [486.1 - 563.3]	-4.00	**<0.0001**
	Accuracy Scores (0 to 1)	0.97 [0.96 - 0.99]	0.98 [0.97 - 0.99]	-4.86	**0.0002**

Cognitive metrics are reported as median [interquartile range]. Mann-Whitney U tests were used to compare concentrations between males and females, and Z-statistics are reported. Significant FDR-corrected p-values are bolded. FDR = false discovery rate, dTMT = digital trail making test, cTST = cued task switching test, ANT = attention networking task. ER = error rate.

Appendix:

MRSinMRS checklist

Lin, A. et al. (2021) Minimum reporting standards for in vivo magnetic resonance spectroscopy (MRSinMRS): Experts' consensus recommendations. *NMR in Biomedicine. 34,* e4484. https://doi.org/10.1002/nbm.4484

**1. Hardware**			
a. Field strength [T]	3 T	3 T	3 T
b. Manufacturer	Philips Healthcare	Philips Healthcare	Philips Healthcare
c. Model (software version if available)	Achieva	Ingenia	Ingenia Elition
d. RF coils: nuclei (transmit/receive), number of channels, type, body part	SENSE 32-channel, phased-array head coil	dStream 32-channel phased-array head coil	dStream 32-channel phased-array head coil
e. Additional hardware			

**2. Acquisition**	
a. Pulse sequence	Point-resolved spectroscopy (PRESS)
b. Volume of interest (VOI) locations	Left primary motor cortex; rostral anterior cingulate cortex
c. Nominal VOI size [cm^3, mm^3]	20 mm isotropic voxel size for both voxels
d. Repetition time (T_R), echo time (T_E) [ms, s]	T_R = 2000 ms, T_E = 30 ms
e. Total number of excitations or acquisitions per spectrum<br>In time series for kinetic studies<br>i. Number of averaged spectra (NA) per time point<br>ii. Averaging method (e.g. block-wise or moving average)<br>iii. Total number of spectra (acquired/in time series)	96 excitations for water suppressed;<br>16 excitations for non-water suppressed
f. Additional sequence parameters (bandwidth in Hz or dwell time in ms, number of spectral points, frequency offsets)<br>If STEAM: mixing time (T_M)<br>If MRSI: 2D or 3D, FOV in all directions, matrix size, acceleration factors, sampling method	2000 Hz spectral bandwidth, 1024 complex data points, 90° flip angle
g. Water suppression method	Vendor-supplied water suppression using a chemical shift selective saturation (CHESS) sequence

h. Shimming method, reference peak, and thresholds for "acceptance of shim" chosen	Vendor supplied "PB-auto", water reference peak
i. Triggering or motion correction method (Respiratory, peripheral, cardiac triggering, incl. device used and delays)	None

**3. Data analysis methods and outputs**	
a. Analysis software	LCModel v6.3-1*R*
b. Processing steps deviating from quoted reference or product	None
c. Output measure (eg absolute concentration, institutional units, ratio), processing steps deviating from quoted reference or product	Absolute metabolite concentrations, reported in mM (institutional units), were corrected for cerebrospinal fluid fraction and T_1 and T_2 relaxation times
d. Quantification references and assumptions, fitting model assumptions	The basis set included alanine, aspartic acid, creatine, phosphocreatine, GABA, glucose, glutamine, glutamate, glycerophosphocholine, phosphocholine, glutathione, myo-inositol, lactate, *N*-acetylaspartate, *N*-acetyl-aspartyl-glutamate, scyllo-inositol, taurine, Lip13a, Lip13b, Lip09, Lip20, MM09, MM12, MM14, MM17, MM20

**4. Data quality**	
a. Reported variables (SNR, linewidth (with reference peaks))	SNR and linewidth
b. Data exclusion criteria	Visual inspection of spectral quality
c. Quality measures of postprocessing model fitting (e.g. CRLB, goodness of fit, SD of residual)	Cramer-Rao Lower Bounds ≤20% were included (threshold set *a priori*)
d. Sample spectrum	Figure 3
